# A Comparison of transgenic and wild type soybean seeds: analysis of transcriptome profiles using RNA-Seq

**DOI:** 10.1186/s12896-015-0207-z

**Published:** 2015-10-01

**Authors:** Kevin C. Lambirth, Adam M. Whaley, Ivory C. Blakley, Jessica A. Schlueter, Kenneth L. Bost, Ann E. Loraine, Kenneth J. Piller

**Affiliations:** Department of Biological Sciences, University of North Carolina at Charlotte, Charlotte, NC 28223 USA; Department of Bioinformatics and Genomics, University of North Carolina at Charlotte, Charlotte, NC 28223 USA; Department of Bioinformatics and Genomics, University of North Carolina at Charlotte, North Carolina Research Campus, Kannapolis, NC 28081 USA

**Keywords:** Transcriptomics, Biotechnology, Pharmaceuticals, Gene expression, Next generation sequencing, Biologics, Glycine max, Equivalence

## Abstract

**Background:**

Soybean (*Glycine max*) has been bred for thousands of years to produce seeds rich in protein for human and animal consumption, making them an appealing bioreactor for producing valuable recombinant proteins at high levels. However, the effects of expressing recombinant protein at high levels on bean physiology are not well understood. To address this, we investigated whether gene expression within transgenic soybean seed tissue is altered when large amounts of recombinant proteins are being produced and stored exclusively in the seeds. We used RNA-Seq to survey gene expression in three transgenic soybean lines expressing recombinant protein at levels representing up to 1.61 % of total protein in seed tissues. The three lines included: ST77, expressing human thyroglobulin protein (hTG), ST111, expressing human myelin basic protein (hMBP), and 764, expressing a mutant, nontoxic form of a staphylococcal subunit vaccine protein (mSEB). All lines selected for analysis were homozygous and contained a single copy of the transgene.

**Methods:**

Each transgenic soybean seed was screened for transgene presence and recombinant protein expression via PCR and western blotting.  Whole seed mRNA was extracted and cDNA libraries constructed for Illumina sequencing.  Following alignment to the soybean reference genome, differential gene expression analysis was conducted using edgeR and cufflinks.  Functional analysis of differentially expressed genes was carried out using the gene ontology analysis tool AgriGO.

**Results:**

The transcriptomes of nine seeds from each transgenic line were sequenced and compared with wild type seeds. Native soybean gene expression was significantly altered in line 764 (mSEB) with more than 3000 genes being upregulated or downregulated. ST77 (hTG) and ST111 (hMBP) had significantly less differences with 52 and 307 differentially expressed genes respectively. Gene ontology enrichment analysis found that the upregulated genes in the 764 line were annotated with functions related to endopeptidase inhibitors and protein synthesis, but suppressed expression of genes annotated to the nuclear pore and to protein transport. No significant gene ontology terms were detected in ST77, and only a few genes involved in photosynthesis and thylakoid functions were downregulated in ST111. Despite these differences, transgenic plants and seeds appeared phenotypically similar to non-transgenic controls. There was no correlation between recombinant protein expression level and the quantity of differentially expressed genes detected.

**Conclusions:**

Measurable unscripted gene expression changes were detected in the seed transcriptomes of all three transgenic soybean lines analyzed, with line 764 being substantially altered. Differences detected at the transcript level may be due to T-DNA insert locations, random mutations following transformation or direct effects of the recombinant protein itself, or a combination of these. The physiological consequences of such changes remain unknown.

**Electronic supplementary material:**

The online version of this article (doi:10.1186/s12896-015-0207-z) contains supplementary material, which is available to authorized users.

## Background

Soybean *(Glycine max)* has been a staple crop and important source of protein worldwide for centuries. The significance of soybean is magnified by the composition of the seed which is naturally rich in protein, oil and linolenic acid [1]. Furthermore, the high protein content of soy (~38 % of dry mass) makes this tissue a fitting candidate for targeted expression of recombinant proteins. The first commercial transgenic soybean plants entered the marketplace in 1996 and contained a gene conferring resistance to the herbicide Roundup. Over the past two decades, a variety of transgenes have been introduced into soy to generate soybeans with increased nutritional content as well as resistance to pests and adverse environmental conditions [2].

In recent years, emphasis on biotechnology has directed many efforts to the generation of genetically modified plants, due in part to the increase in their potential for applications in the pharmaceutical industry. With increasing healthcare costs and shortages of medication alternatives, there has been much interest in the development of cost-effective biologics. Proteins have been generated in bulk via bacterially derived methods for years, but limitations in protein size and post-transcriptional modifications have demanded the development and use of other expression systems. Traditional eukaryotic expression systems such as yeast, insect and mammalian cell cultures remedy many of these issues, but production costs of protein purification and storage usually proves to be expensive [2–4]. Plant systems have proven to be an economically viable alternative to cell culture systems, despite involving more complex molecular and genetic design phases prior to transformation. Although *Arabidopsis* and tobacco represent heavily utilized model plant systems, they require sizeable quantities of leaf biomass for extracting large quantities of recombinant protein.

Soybeans represent one of the richest natural sources of protein on a per mass basis. Soybean seeds represent a favorable biochemical environment for production of large and complex proteins that are often recalcitrant to expression in traditional systems [5]. Furthermore, transgenic soybeans can be stored as ground powder for years without a need for refrigeration [2, 6, 7]. For these reasons, our laboratory has been interested in developing soybean as a platform for the expression of cost-effective therapeutics [2, 5, 8] that can either be purified or formulated for oral delivery [2, 9]. Although soybean transformation is technically challenging and requires lengthy regeneration times, once transgenic events have been generated and taken to homozygosity they represent a low cost, sustainable solution for production of recombinant protein [10]. Our laboratory has successfully expressed a variety of recombinant proteins in soybean seeds, including subunit vaccines for traditional injection and oral delivery [9, 11, 12], immunogens for treatment of autoimmune disease, and diagnostic reagents for the detection of cancer [5, 8]. The production of these novel soy-based proteins have the potential to address current unmet needs in the healthcare industry and provide novel processing, formulation, and delivery options of therapeutics that are not currently available. Our group and others [13] have reported the expression and accumulation of recombinant proteins in soybean to levels approaching 3 % of total soluble seed protein. These levels equate to >1 mg target protein per seed and represent a significant yield of target protein contained within an environmentally stable package. The production of such large quantities of recombinant protein raises fundamental questions regarding the transcriptional profiles and proteomics in transgenic seeds.

Transgenic plants have been investigated for comparative equivalence to their wild type derivatives prior to deregulation of commercial crops to ensure that the inserted transgene does not negatively impact the quality and nutritional value of seeds and grains [14]. Typical analyses of “substantial equivalence” for transgenic plants stems from the FDA guidelines for inspection, and have traditionally used metabolites, antioxidants, oils, and other molecular compositions as measurements for equivalency [15, 16]. Studies in crop species and other edible plants have determined that compositional variation is typically within the natural range observed through traditional breeding methods [17–21]. While most studies conclude that measured differences are insignificant, some nutritional and metabolic differences have been observed in different transgenic events [22–24]. Such studies conducted using transgenic soybean have shown only minor fluctuations in metabolites, free amino acids and sugar content, but surprisingly demonstrate that seed protein content remains unchanged [25–27]. Although acceptable levels of variance have not been clearly defined for specific molecules, significant differences from wild type organisms in the above mentioned studies have not been demonstrated in the examined plants, or shown to have long-term health impacts when used for human consumption [21, 28].

Due to the random nature of the mechanisms associated with plant transformation [29], transgene cassettes could integrate at genomic locations that may positively or negatively impact recombinant protein expression and accumulation [30]. Insertion could also affect the expression of neighboring and downstream genes from the insertion site. Due to the myriad of feedback mechanisms associated with gene expression and regulation, it is possible that disruption of a single exon could alter expression of hundreds or thousands of other genes. Comparative analyses of genetically modified plants has been previously conducted [21], however those studies focused on metabolomics, proteomics and nutritional comparisons. For years genomics and transcriptomics have been recommended as additional evaluation criteria for inclusion in substantial equivalence studies [31]. In this regard, microarrays have been utilized to examine differences between transgenic plants and their wild type equivalents [17] and to detect differentially expressed genes under a variety of environmental conditions.

Recent developments in next generation sequencing technologies, in conjunction with the publication of the soybean genome and transcriptome, allows access to more detailed information and refined tools that were not previously available, which in turn can lead to more accurate detection of differentially expressed genes. In this study, we utilized the most recent sequencing technology available on the Illumina platform to conduct whole transcriptome sequencing of seed tissue from three soybean lines developed in our laboratory. These lines express three different recombinant proteins that accumulate to varying levels, with ST77 expressing hTG at 1.61 %, 764 expressing mSEB at 0.76 %, and ST111 expressing MBP sigma at 0.07 % of total soluble protein. The resulting datasets were used for direct transcriptomic comparisons with identically treated wild type seeds. We found that varying numbers of genes were differentially regulated in all three transgenic soybean lines, with one line having significantly more extensive differences than the others. These results demonstrate the potential for significant transcriptomic variances in transgenic events. To our knowledge, this study represents one of the first to compare the transcriptomes of transgenic soybean seeds with their wild type counterparts using significant statistical power and reproducibility.

## Methods

### Vector construction and transformation of soybean

The binary constructs used to generate the 764 events expressing mSEB protein and ST77 events expressing hTG protein have been previously described by our laboratory [5, 11]. The binary construct used to generate the ST111 events was similar in design to the ST77 binary vector with the exception of the target gene, which encodes a novel fusion protein referred to as hMBP-Sigma. A soybean codon-optimized synthetic gene encoding hMBP-Sigma was synthesized by DNA2.0 (Menlo Park, CA). This gene contained sequences encoding the soybean glycinin signal peptide and full-length myelin basic protein fused to the Reovirus Sigma 1 protein [32]. The hMBP-Sigma fusion protein was engineered with NcoI and XbaI restriction endonuclease sites at the 5’ and 3’ termini respectively, to facilitate cloning. To generate the ST111 binary vector, the ST77 binary vector was digested with NcoI and XbaI (to release the hTG coding region) and the resulting vector backbone was ligated with the synthesized hMBP-Sigma gene that was also previously digested with NcoI and XbaI. The resulting ST111 binary vector used for soybean transformation contained the 7S β-conglycinin promoter, Tobacco Etch Virus (TEV) translational enhancer, glycinin signal peptide, hMBP-Sigma fusion protein and the 35 s terminator. The ST111 binary vector also contained a selectable marker cassette utilizing the phosphinothricin acetyltransferase (BAR) gene under control of the nopaline synthase (NOS) promoter and terminator sequences. The integrity of ST111 was verified by multiple restriction digest analyses and double-stranded sequencing of the hMBP-Sigma gene (Davis Sequencing, LLC, Davis CA). Transformation of soybean (Williams 82) was performed using the cotyledonary-node *Agrobacterium-*mediated half-seed method previously described [33]. The Williams 82 cultivar of soybean is the same cultivar used for the release of the soybean genome [34]. The declaration of rDNA constructs and propagation of transgenic soybeans was approved by the University of North Carolina at Charlotte Institutional Biosafety Committee.

### Soybean cultivation

**Fig. 1:**
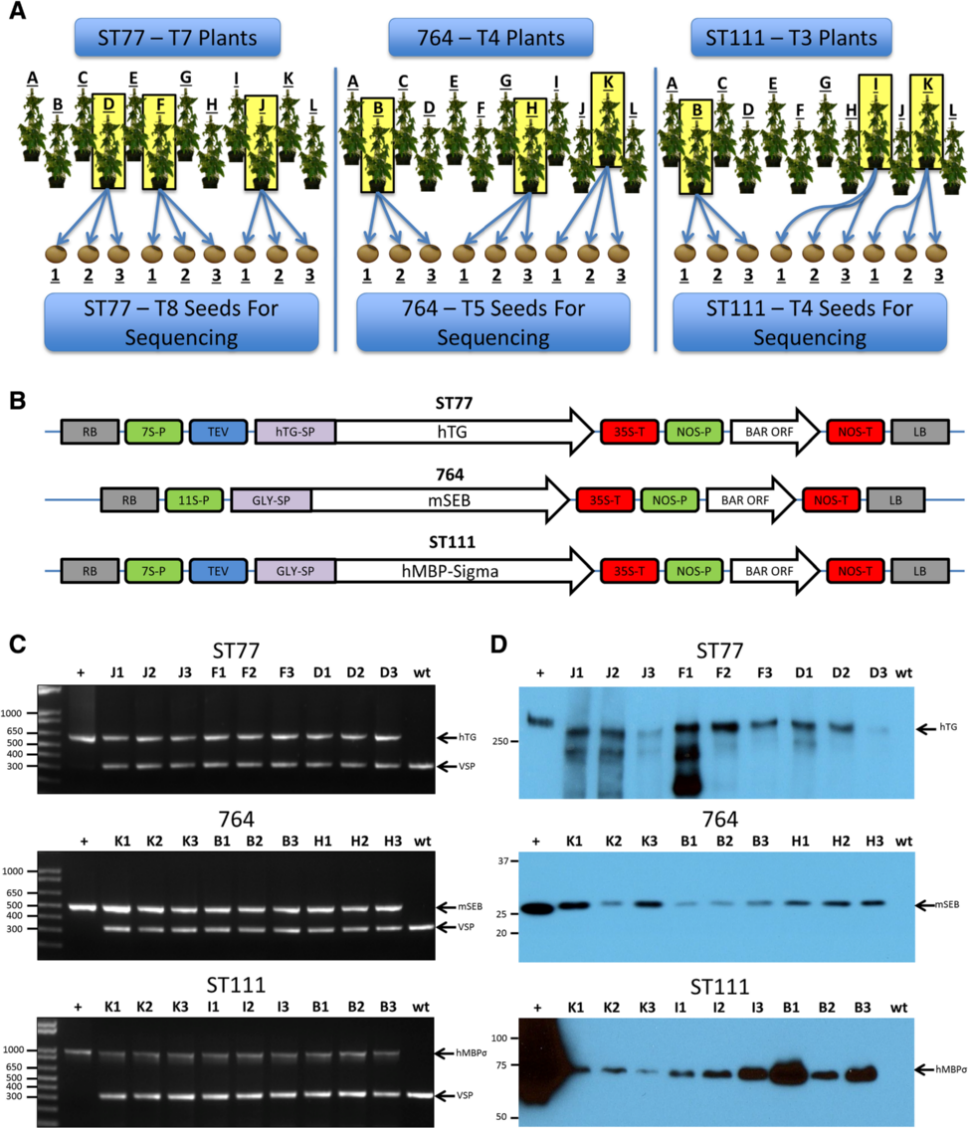
Experimental design and gene constructs. (**a**) The selection and propagation process of the plants and seeds used in this study. (**b**) The binary vectors used for *Agrobacterium*-mediated transformation are shown. The regulatory elements include: 7S-P (7S soybean *β*-conglycinin promoter), TEV (tobacco etch virus translational enhancer element), hTG (human thyroglobulin gene), hTG-SP (hTG signal peptide), 35S-T (35S cauliflower mosaic virus terminator element), Gly-SP (soybean glycinin signal peptide), hMBP-Sigma (human myelin basic protein fused to Reovirus Sigma 1 protein), 11S-P (soybean 11S glycinin promoter), mSEB (mutant nontoxic staphylococcal enterotoxin B gene), NOS-P (nopaline synthase promoter), BAR (phosphinothricin acetyltransferase gene) and NOS-T (nopaline synthase terminator element). Arrows indicate orientation of cassettes relative to the right border (RB) and left border (LB) sequences. Regulatory elements and genes are not drawn to scale. Molecular characterization of transgenic events. (**c**) Duplex PCR of the nine progeny seeds from the indicated transformation events. wt: nontransgenic (negative control); +: plasmid DNA (positive control). Arrows indicate amplified DNA fragments derived from the specific gene of interest as well as vegetative storage protein gene (VSP) following separation in agarose gels. Sizes of molecular weight markers are shown in base pairs. (**d**) Western blots of total seed protein derived from the transgenic progenies shown in (**c**). Arrows indicate the hTG, mSEB and hMBP-Sigma immunoreactive proteins. Sizes of molecular weight standards are shown as kDa. Positive controls (+) are purified hTG (Cal Biochem), *E. coli-*derived mSEB, and soy-derived hMBP-Sigma from a higher expressing line

Seeds from each transgenic event as well as from wild type were germinated in moistened soil in 6-pack planting trays. Following germination, plants were propagated in Scott’s 6-month nutrient Miracle Grow potting mix with 16 h light (26 °C) and 8 h night cycles (20 °C) in controlled growth chambers with ~50 % relative humidity. Plants were watered every other day or as needed if the soil was observed to be dry, and were transferred to 4-in. pots (Dillen Greenhouse, 4.00 Square Traditional) at 3 weeks of age and then 1.5 gal pots (Nursery Supplies Inc. C600) at 6 weeks of age. Light intensities were measured at ~500–550 μE m^−2^s^−1^. Three plants were chosen from each genotype, which were all phenotypically identical to wild type plants with respect to overall size, leaf structure, and approximate seed yield. Dried pods were collected following senescence and fully matured dry seeds at the final R8 stage of development were removed and used for molecular characterization and transcriptome sequencing. Three seeds from each plant were collected and processed individually, generating three biological replicates from each plant, and three biological replicates from each construct. ST77, 764, and ST111 seeds were obtained from T7, T4, and T3 generation transgenic plants respectively, and were stored in individual seed bags at 23 °C and 50 % relative humidity until processing. In total, nine seeds were chosen from each transgenic event and from wild type for a total of 36 samples (See Fig. 1a).

### Transgenic soybean genomic dna extraction and duplex PCR

Genomic DNA was extracted from seed cotyledon tissue using a Maxwell 16 Instrument and DNA extraction kit (Promega, Madison WI) and cleaned by phenol-chloroform extraction followed by ethanol precipitation. Duplex PCR conditions for ST77 and 764 were described previously [5, 11]. For ST111 duplex PCR, ~1 μg of genomic DNA was mixed with GoTaq Flexi DNA polymerase (Promega, Madison, WI) and buffers provided by the manufacturer with the following primers: hMBP forward (5’-ATGGACCCAAGACTTAGAGAGG-3’), hMBP reverse (5’-CCACATAGACTGTCTGAACCTG-3’), vegetative storage protein (VSP) forward (5’-GCTTCCACACATGGGAGCAG-3’), and VSP reverse (5’-CCACATAGACTGTCTGAACCTG-3’). Following an initial 5-min denaturation step at 95 °C, amplification was performed using 38 cycles of denaturation (95 °C for 30 s), annealing (50 °C for 45 s), and extension (72 °C for 60 s), followed by a final extension step (72 °C for 5 min). Amplified products were separated and visualized in 1.0 % agarose gels.

### Transgenic soybean seed protein extraction and western blot analysis

Seed protein was extracted and quantified as previously described [12]. Briefly, sections of cotyledon tissue from mature seeds were placed in 300 μL of phosphate-buffered saline and sonicated for ~15 s. Samples were centrifuged to clarify soluble protein from insoluble debris, and the clarified protein was quantified using a Bradford assay (Bio-Rad, Hercules CA) with bovine serum albumin (BSA) as a standard. Due to the various sizes and inherent properties of each recombinant protein, a variety of different polyacrylamide gel concentrations and buffers were used for the separation of proteins prior to immuno-detection. For analysis of hTG protein, 5 μg of ST77 seed protein extracts were separated in 5 % native SDS gels using non-reducing conditions as described previously [5]. For analysis of mSEB protein, 3 μg of 764 seed protein extracts were separated in 10 % SDS gels using standard reducing conditions as previously described [11]. For analysis of hMBP-Sigma protein, 20 μg of ST111 total seed protein extract was incubated with non-reducing sample buffer (10 μg of bromophenol blue, 3 % SDS, 1.5 % glycerol, and 0.025 M Tris–HCl) and separated in 8 % SDS-PAGE gels. Following electrophoresis at 100v for ~2 h, gels were incubated with 1× CAPS buffer (3-[Cyclohexylamino]-1-propanesulfonic acid) in 10 % methanol and transferred to nitrocellulose Immobilon P membranes (Millipore, Billerica MA) for 1 h at 100 v. Membranes containing transferred protein were blocked in 1× PBS containing 5 % non-fat milk powder overnight at 4 °C, followed by a 3-h incubation at 23 °C with respective primary antibodies. Blots were washed three times for 15 min each in 1× PBS/0.1 % SDS and incubated with a secondary antibody (HRP-linked goat anti-rabbit IgG) for 1 h at 23 °C. Blots were washed again three times for 15 min each in 1× PBS/0.1 % SDS prior to a 5-min incubation with 10 mL of SuperSignal West Pico luminol enhancer solution (Thermo Scientific, Rockford, IL) at 23 °C before detection with film.

### RNA Extraction

Each of the selected seeds was cut in half along the embryonic axis using an RNase-free razor. To eliminate possible RNA contamination, RNaseOUT (G-Biosciences, St. Louis MO) was used throughout the extraction procedure. Bisected seed halves including the testa, hilum, micropyle, and embryo tissue were flash frozen in liquid nitrogen and ground to a fine powder with a mortar and pestle. Crushed powder was immediately transferred to RNase/DNase-free 1.5 mL spin tubes. Total RNA was extracted and purified using the RNeasy Plant Mini Kit protocol (Qiagen, Germantown MD) for plant cells and filamentous fungi. Buffer RLC was incorporated as recommended by the protocol due to the high concentrations of starch and metabolites in soybean seed tissues. Residual DNA contamination was removed by treating the spin column with 30 units of RNase-free DNase I (Invitrogen, Grand Island NY) for 15 min at 23 °C prior to RNA elution. RNA concentrations and purity were verified for each sample following elution with a Nanodrop 2000 spectrophotometer (Thermo Scientific, Waltham MA). The 260/280 nm wavelength ratios were ~2.0 for all samples with an RNA concentration ranging from 0.1-1.0 μg/μL. RNA samples were stored at -80 °C for up to two weeks until all cDNA libraries were prepared.

### Library construction

cDNA libraries for each sample were generated using the TruSeq RNA Sample Preparation Kit A (Illumina, San Diego CA) according to the recommended low-sample TruSeq RNA Sample Preparation Guide protocol (Illumina, version 2 revision C). Samples were prepared in four groups, with nine samples per event for a total of 36 libraries. 50 μL of total RNA was loaded into 0.2 mL DNase and RNase-free PCR tubes for use during the purification steps prior to amplification. cDNA was generated through reverse transcriptase PCR using Superscript II reverse transcriptase (Invitrogen, Carlsbad CA). cDNA was bound for purification during the protocol with Agencourt AMPure XP beads (Beckman-Coulter, Pasadena CA). Following ligation of unique adapter sequences, the DNA was enriched by PCR with 15 cycles of amplification according to the TruSeq protocol. Ligation and library integrity was verified using a DNA chip on the Agilent 2100 Bioanalyzer (Agilent, Santa Clara CA) with clean elution profiles at the correct size peak of 261 bp. The Illumina TruSeq kit “A” adapter sequences were ligated to each sample in each group to allow for sequencing multiplexing (see Additional file 2: Table S1). Samples were stored at -20 ° C for up to 2 weeks until single-end sequencing could be conducted on all samples simultaneously.

### Sequencing

Sample libraries ligated with unique adapter sequences were multiplexed six to a lane and were sequenced by the David H. Murdock Research Institute Core lab genomics department (Kannapolis, NC) using Illumina HiSeq 2000 100-cycle, single-end sequencing. Additional file 2: Table S1 reports ligated adapter sequences and other details of sequencing strategy and multiplexing. Quality control analysis on the resulting fastq sequencing files was performed using FastQC (Babraham Bioinformatics, Cambridgeshire UK). FastQC reports for each sequence file are available in the project repository in the folder named “FastQC”.

### Sequence alignment

Sequence reads were aligned onto soybean transcriptome and genome reference sequences using tophat version 2.0.13 [35] using the maximum intron size (-I) parameter 5000 as recommended for non-mammalian genomes. Gene structure annotations corresponding to the latest annotation release were used to build a transcriptome index and provided to tophat during the alignment step. A copy of the gene annotations was obtained from the Joint Genome Institute (JGI) [36] download site for soybean and is version-controlled in the project repository in the “ExternalDataSets” folder. The reference genome used was version 2.75 [37] supplemented with scaffolds containing transgene sequences. Sequence files are available from the Short Read Archive [38] under accession SRP051659.

### Differential expression analysis with edgeR

The featureCounts program [39] was used to count the number of reads aligning to annotated soybean genes and the transgenes. The program was invoked three times with different options to enable different treatment of reads with ambiguous genomic mappings. The “sm” (single-map) invocation ran featureCounts with default settings ensuring only single-mapping reads were counted. The “mm” (multi-map) invocation added the option “-M”, which counted read alignments for reads with more than one alignment. The “pm” invocation added the option “--primary”, which counted just the primary alignments for reads, including reads that mapped multiple times but ignoring alignments not reported as a primary alignment for a read. Files produced by featureCounts, including both outputs and summary reports, are available from the project repository in the “data” subfolder within the “Counts” directory. Since comparing pm and mm files indicated that the results were similar (see the file CountsComparison.html in the “Counts” folder), only the pm gene counts were used in subsequent differential expression analyses. Expression values in reads per million (RPM) and reads per kilobase transcript per million (RPKM) were calculated for the sm and pm data sets and are also available in the “results” subfolder within the “Counts” directory.

EdgeR [40] version 3.8.5 was used to identify differentially expressed genes. Following the procedures described in the edgeR documentation, read count tables were loaded into R, normalized using the default method for edgeR (trimmed mean of M values, or TMM), and then tested for differential expression using the exactTest method. P values reported by edgeR were used to calculate false discovery rates (FDR) for each gene using the method of Benjamini and Hochberg [41]. Results from differential testing of every gene are available in the results directory of the “DiffExpr” folder in the project git repository. Fold-changes are reported as the log (base 2) of normalized count abundance of the transgenic samples divided by count abundance for the wild type (nontransgenic samples). Samples were clustered by the differentially expressed gene lists using multi-dimensional scaling (MDS) plots (Additional file 3: Figure S1A-C), and were also grouped according to all detected genes in dendrograms (Additional file 3: Figure S1D-F).

### Differential expression analysis using cufflinks

Cufflinks version 2.2.1 [42] was used in addition to edgeR as a complementary approach to differential expression analysis. Read mapping was performed as described above and reads were assembled using cufflinks*,* including parameters for fragment bias correction and multi-read correction. Scripts used to run cufflinks are in the project repository in the folder named “src” at the top of the source code tree. The resulting output was used to create a merged GFF file using cuffmerge, and this merged GFF was used in the differential expression analysis with cuffdiff, again using multi-read and fragment bias correction parameters (see Additional file 4: Figure S2). Fold-changes are reported as the log (base 2) of normalized read count abundance for the wild type samples divided by the read count abundance of the transgenic samples. Output of cufflinks in the form of GTF files are available from the Gene Expression Omnibus [43] under accession number [GEO:GSE64620]. Version-controlled data processing and analysis code are available from the project git repository at http://bitbucket.org/lorainelab/soyseq.

### Gene ontology analysis

As in the differential expression analyses described above, gene ontology (GO) enrichment analysis was conducted twice in parallel for each transgenic line. In both methods, only genes with a FDR 0.01 or smaller were considered for the 764 line. For analysis of ST111 and ST77 lines, a FDR of 0.05 was used. The DE genes list for both GOseq and AgriGO included all DE genes (genes called as DE by edgeR or cuffdiff).

The GOseq package version 1.18.0 [44] was used to identify GO categories with unusually many or unusually few differentially expressed genes in the merged dataset. Categories with unusually many differentially expressed genes represented functions, processes, or cellular components that were affected by the transgene, while categories with unusually few differentially expressed genes represented processes that were resistant to perturbation by the transgene. GOseq was used in order to correct for well-known bias in which differentially expressed genes with larger transcripts are easier to detect. GO annotations for soybean were from the “annotation info” file downloaded from the JGI Web site and version-controlled in the “ExternalDataSets” folder of the project repository. Code used to run the analysis resides in the folder named “GeneOntologyAnalysis” in the project repository.

Upregulated and downregulated genes from the merged DE edgeR and cufflinks output were also loaded into the AgriGO web tool [45] to identify enriched GO terms for visualization and to complement the GOseq results. Each list was entered into a single enrichment analysis (SEA) against the current *Glycine max* background reference provided by Phytozome [37] using a Fisher’s exact statistical test and Hochberg FDR post-hoc test.

### Availability of supporting data

Sequence files supporting the results of this article are available from the NCBI Sequence Read Archive [38] under accession number [SRP051659]. Output of cufflinks in the form of GTF files are available from the Gene Expression Omnibus [43] under the accession number [GSE64620] available at [http://www.ncbi.nlm.nih.gov/geo/query/acc.cgi?acc=GSE64620]. Version-controlled data processing and analysis code are available from the project git repository [http://bitbucket.org/lorainelab/soyseq]. Analysis code available in the repository includes shell scripts used to run data processing programs and R Markdown files used to perform statistical analysis. R Markdown output files (with file extension “HTML”) documenting the details of analysis are available and can be opened and examined using a web browser. R markdown output files contain version numbers of all R libraries used. Additional instructions for viewing analysis results and data are available on-line at the project repository web site. Alignments from tophat, coverage graphs, and assembled reads (from cufflinks) are available for visualization in Integrated Genome Browser [46] from the IGBQuickLoad site [http://igbquickload.org/soy].

## Results

For this study we chose seed tissue derived from three independent transgenic lines expressing different recombinant proteins at varying levels of accumulation. All three lines were generated using *Agrobacterium-*mediated transformation methods. A summary of the selection process is shown in Fig. 1a, and the binary vectors used to create these transgenic lines are shown in Fig. 1b. ST77 is a transgenic line expressing the 330 kDa human thyroglobulin protein (hTG); ST111 is a transgenic line expressing a 75 kDa protein comprising the human myelin basic protein fused in frame to the Reovirus Sigma1 protein (hMBP-Sigma); and 764 is a transgenic line expressing a 28 kDa mutant, nontoxic form of a staphylococcal subunit vaccine protein (mSEB). All three lines are homozygous with a single T-DNA insert at a single genomic locus. While ST111 was originally a complex insertion event containing T-DNA insertions at multiple loci, segregation of loci from multiple generations resulted in the single copy line used for these studies. Southern blot screens were used to characterize complexity of all lines (data not shown). For biological replicates, three plants were chosen from each transgenic line, and three seeds were selected from each plant (Fig. 1a). In the same fashion, three seeds from three wild type parents were also chosen for a total of nine individual negative controls.

### Molecular analysis and sequencing

Prior to Illumina sequencing, molecular analyses were performed to verify the presence of each respective transgene in each seed as well as expression of the corresponding recombinant protein. To assay for transgene integration, duplex PCR was performed using two sets of primers for simultaneous detection of the transgene and internal control gene (vegetative storage protein). The results of these PCR assays are shown in Fig. 1c. In all cases, the presence of stably integrated T-DNA in each seed genome was verified.

Western analyses were carried out to demonstrate the stable accumulation of recombinant protein in each of the selected seeds and these results are shown in Fig. 1d. It should be noted that 3–5 μg of seed protein was sufficient for visualization of hTG and mSEB in lines ST77 and 764, while 20 μg of protein was required for visualization of hMBP-Sigma protein from line ST111. We estimate that recombinant hMBP-Sigma protein accumulates to a level representing 0.07 % of total soluble protein (TSP); therefore ST111 was classified as a line expressing a “low” level of recombinant protein. For comparison, ST77 and 764 are classified as lines expressing relatively “high” and “medium” levels of recombinant protein as hTG accumulates to 1.61 % TSP [5] while mSEB protein accumulates to 0.76 % TSP in the 764 line [11].

**Fig. 2:**
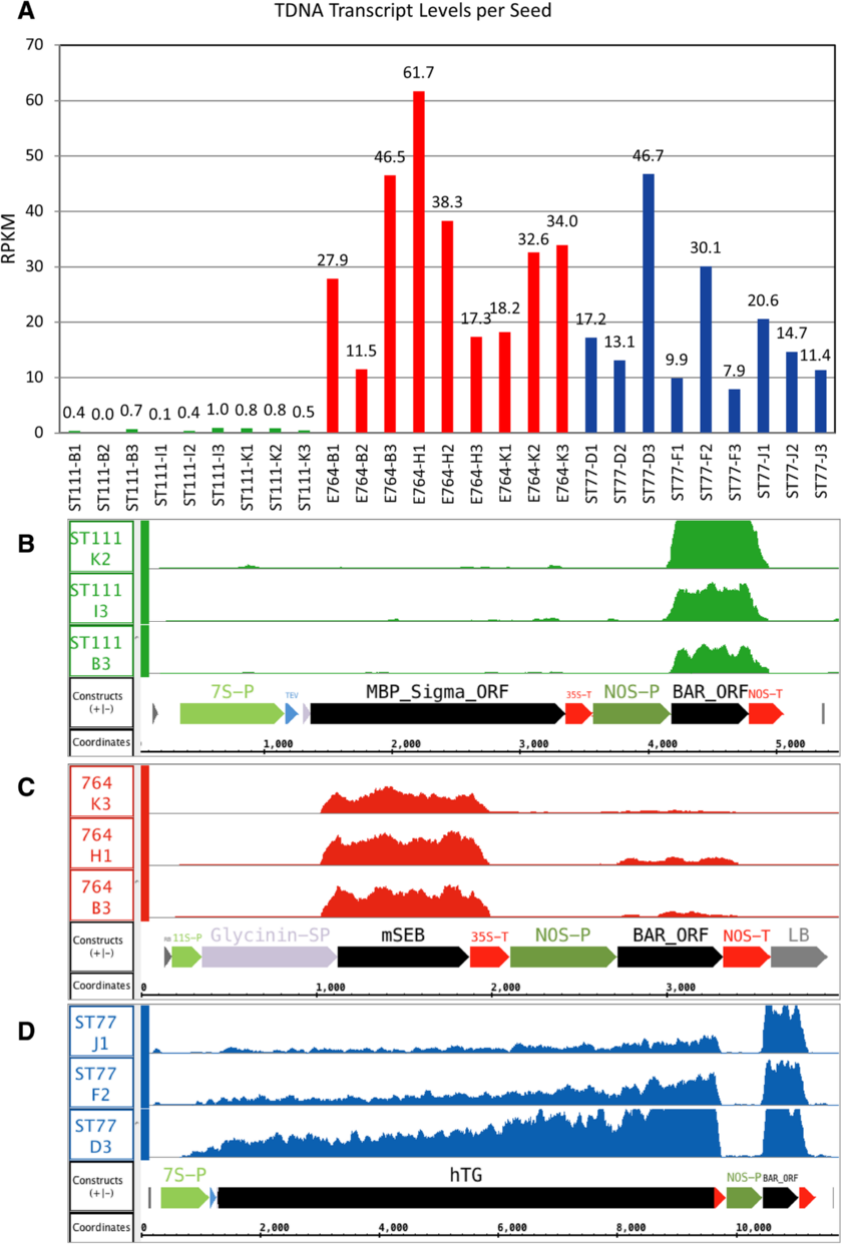
T-DNA transcript levels and coverage in transgenic seeds. (**a**) The number of reads aligned to the T-DNA sequence by Tophat from the reference genome comprising an extra scaffold containing the respective gene of interest cassette. Numbers on the y-axis represent RPKM normalized expression values for the gene of interest in each sample. (**b**-**d**) Coverage graphs over the annotated region of each added scaffold are shown for lines ST111 (**b**), 764 (C), and ST77 (**d**). The annotated components correspond to those shown in Fig. 1. The highest expressing seed is shown for each transgenic plant. The y-axis of each coverage graph ranges from 0 to 100

Illumina sequencing was performed on libraries prepared from seed cDNA from each set of nine transgenic seeds as well as nine wild type seeds of the same genotype. Single-end, 100 base sequencing generated between 7 and 12 million reads per library. Reads were aligned to the reference genome and transcriptome and mRNA expression levels for transgenes and native soybean genes were assessed. Normalized expression values per sample for the transgenes are shown in Fig. 2a. Coverage maps from the highest expressing seed from each transgenic plant are depicted in Fig. 2b–d. The ST111 line which accumulated the least amount of recombinant protein showed the fewest aligned T-DNA reads, while the ST77 and 764 lines expressed greater levels of recombinant protein and showed a higher number of aligned reads. Note that the transcript levels of ST77 and 764 are similar despite ST77 expressing twice as much recombinant protein by mass as 764. This observation is likely due to the large size of the hTG transgene coupled with fewer aligned reads in the upstream portion of the gene, and can be visualized in the coverage maps (Fig. 2c–d). Analysis of the coverage data revealed accurate transcript initiation and termination of each transgene. Similarly, accurate initiation and termination of the selectable marker gene transcripts (BAR) was also observed in all cases.

**Fig. 3:**
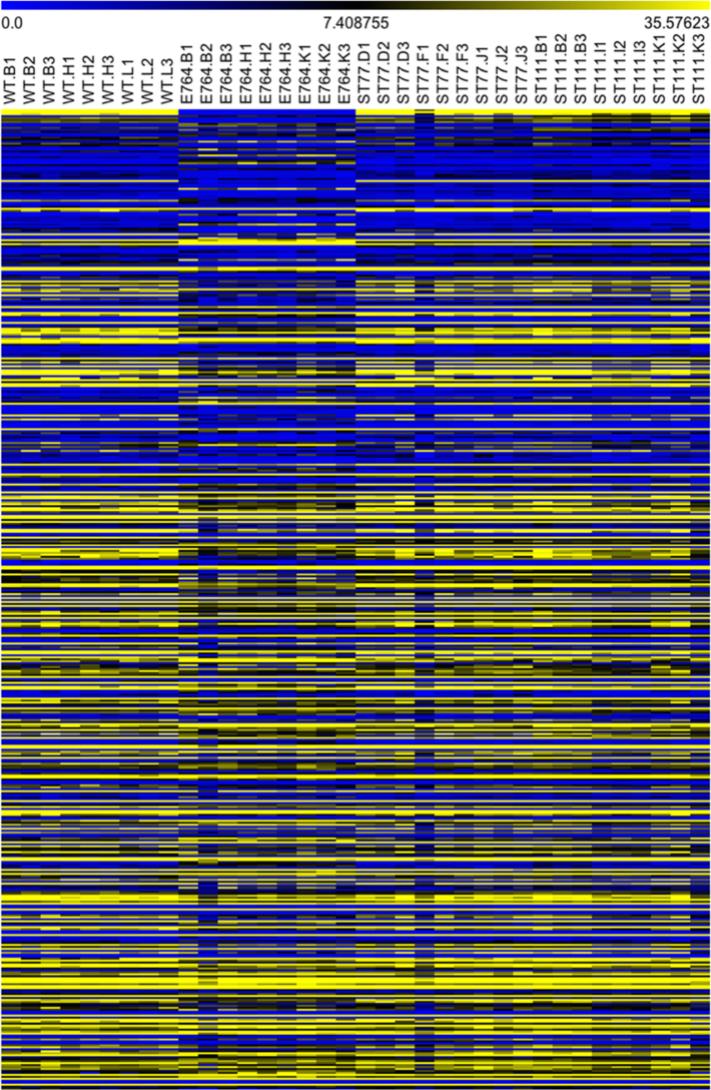
Heatmap generated from the top 500 differentially expressed genes as reported by edgeR. Genes are sorted in descending order according to fold-change. The yellow color indicates higher levels of gene expression while blue indicates lower expression by RPKM

RNA-seq data was analyzed using cuffdiff and edgeR as complementary differential expression analysis methods. Several studies have suggested that combining and comparing outputs from complementary methods such as these can yield more accurate results [47–51]. Using a false discovery rate (FDR) of 0.01, the edgeR-based analysis identified relatively few differentially expressed genes in the ST77 and ST111 lines (52 and 307 respectively), but found ~3800 total up and downregulated genes in the 764 line. To illustrate differences between the lines, a heat map was constructed using TM4 MeV software [52] showing RPKM expression values for 500 of the most differentially expressed genes in the 764 versus nontransgenic comparison (Fig. 3). It should be noted that because ST77 and ST111 only contained 52 and 307 significant differentially expressed genes, transcripts displayed in Fig. 3 beyond these for ST77 and ST111 are sorted by decreasing average detected logfold change for ease of comparison between the three lines. Expression levels of the top differentially expressed genes in the 764 line were different from wild type, ST77, and ST111 gene expression. Expression differences were consistent within all groups with the exception of one outlier in the ST77 group (ST77 F1). It should be noted that the archived seed of sample ST77 F1 showed visible fungal growth two weeks after sequencing; this growth was not visible during the selection process, however, it may be one explanation for the observed differences in expression. The inclusion of ST77 F1 in the analysis did not alter the conclusion that ST77 was the most similar to wild type.

**Fig. 4:**
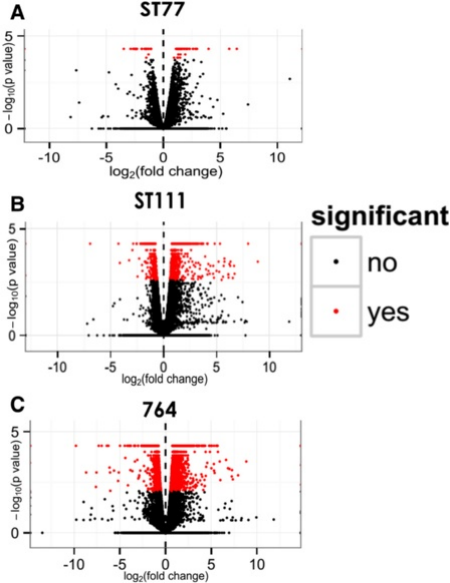
Cufflinks plots of differentially expressed genes. (**a**-**c**) Cufflinks volcano plots for each transgenic event showing variances in gene expression with respect to fold-change and significance. Each dot represents an individual gene. Black dots represent genes that are not significantly differentially expressed while red dots represent genes that are significantly differentially expressed

**Fig. 5:**
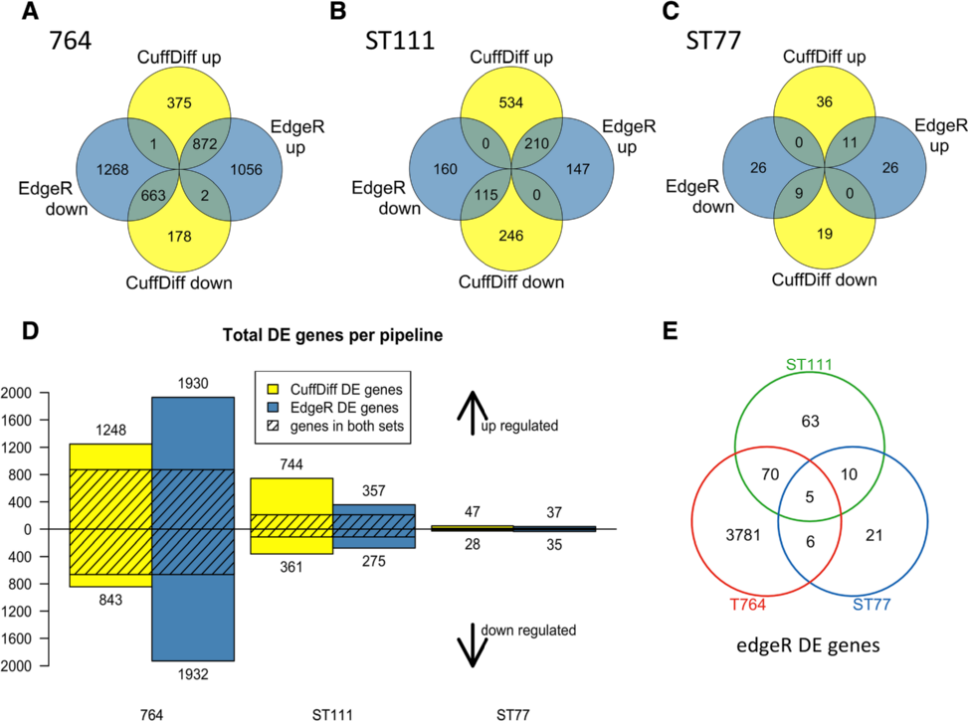
Venn diagrams of differentially expressed genes between edgeR and cufflinks. Numbers of genes that are up and down-regulated in both edgeR and cufflinks are shown for 764 (**a**), ST111 (**b**), and ST77 (**c**) lines. Total differentially expressed genes for each line determined by each program and the number of shared genes for each is shown in (**d**). Numbers of differentially expressed (DE) genes shared between each line from the edgeR results are illustrated in (**e**). Significance was defined by an FDR of 0.01

Cufflinks software was used in addition to edgeR to investigate differential expression and revealed similar differences in gene expression. Cufflinks reported 47 upregulated and 28 downregulated genes in ST77, 744 upregulated and 361 downregulated genes in ST111 and 1249 upregulated and 843 downregulated genes in 764. Volcano plots were constructed from the results and are shown in Fig. 4. These plots show the relationship between fold change and statistical significance of differentially expressed genes. Note that there is >20-fold difference in the number of differentially expressed (DE) genes between ST77 and 764. Thus, it is clear from both the edgeR and cufflinks results that while there were significant differences in all three transgenic events, differences were the most substantial in line 764 relative to the wild type controls. The results of these two programs are illustrated in Fig. 5. The Venn diagrams (Fig. 5a–c) indicate the number of up and downregulated genes identified by each program separately and together, while the bar chart (Fig. 5d) shows the total number of upregulated and downregulated genes as well as the portion of shared genes identified from each program. Five genes were differentially expressed in all three transgenic lines, including Glyma.12G136600 (protein kinase), Glyma.13G171200 (ribosomal RNA protein-7 related), Glyma.01G103100 (branched chain alpha-keto acid decarboxylase E1 beta subunit), and two genes with no functional annotation information (Glyma.07G207000, Glyma.13G011800). Glyma.01G103100 and Glyma.13G171200 showed no commonality between the three events in the direction of altered expression; however Glyma.01G103100, Glyma.07G207000, and Glyma 13.G011800 were upregulated in all three transgenics. Fig. 5e shows the number of common DE genes shared between each of the three lines based on the edgeR results. A list of all shared differentially expressed genes between all events is available in the git repository file “Diffexpoverlap” under the “DiffExp” directory.

**Fig. 6:**
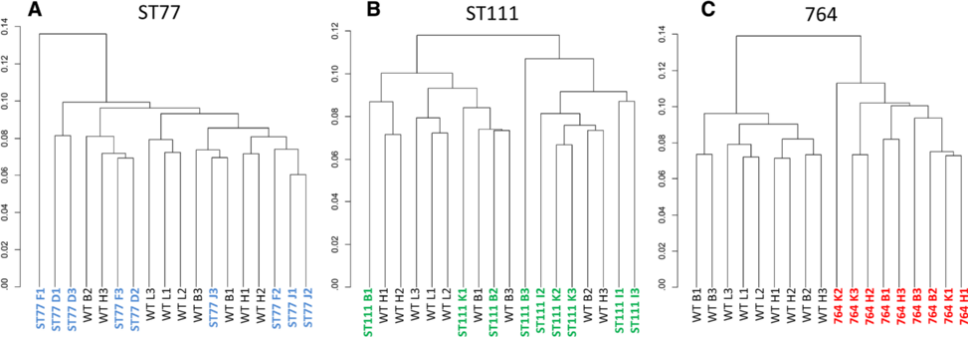
Dendrograms prepared by cummeRbund using differentially expressed gene lists*.* Samples were clustered in their respective groups compared to wild type and plotted based on variance (**a**-**c**)

Numbers of DE genes are a function of statistically significant gene calls within groups, but do not illustrate between sample variance. Clustering algorithms integrated in cummeRbund allowed visualization of individual sample similarity and variance in comparison to wild type by generating dendrograms with the “csdendro” command. Dendrograms allow visualization of between sample variance, reflected by their clade distance from others. Based on the differentially expressed gene sets, the ST77 and ST111 samples clustered randomly intermixing with wild type samples, while the 764 samples clustered independently of wild type (Fig. 6). ST77 and ST111 samples clustered across both their respective biological groups and the wild type group showing variances were not substantial enough to completely segregate, while all 764 samples were in a distinct clade from wild type.

### Gene ontology results for 764 using GOseq

**Fig. 7:**
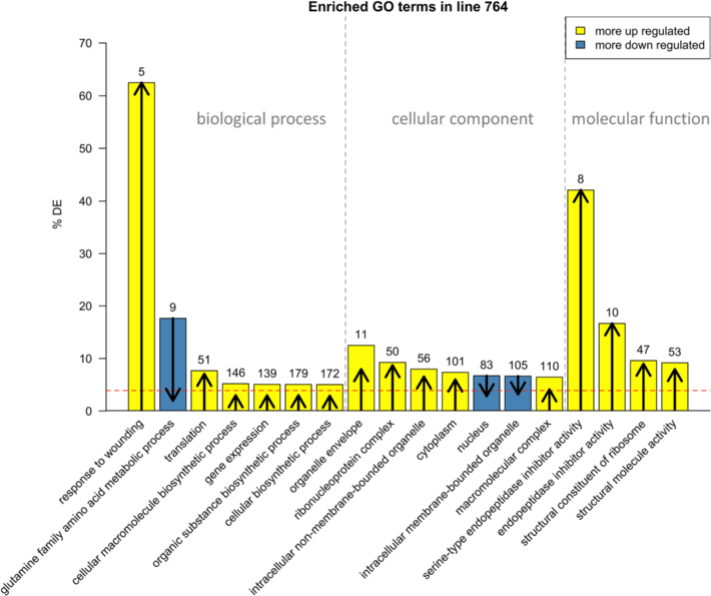
GOseq results from the edgeR and cufflinks merged DE gene list. The numbers over each bar indicate the total number of differentially expressed (DE) genes in each category. The length and direction of the arrow in each bar indicate how many of the genes were up (arrows pointing up) or down (arrows pointing down) regulated in that category. The red dashed line indicates the percentage of all GO-annotated genes that are DE from edgeR and cufflinks in this line (~4 %). All terms shown have an FDR of 0.05 or smaller

We next performed a gene ontology (GO) enrichment analysis using GOseq [44] which accounts for selection biases in RNA-Seq data in which larger, more highly expressed transcripts are preferentially detected as differentially expressed. In line 764 we detected at least one read sequence from ~42,000 of the 56,000 annotated soybean genes, and of these ~42,000 expressed genes, approximately 3800 (9 %) were differentially expressed. The input list consisted of approximately 1500 genes that were considered differentially expressed after combining the lists from both edgeR and cufflinks. Thus, on average, we expected that approximately 3.5 % of genes in any random sample of expressed genes would be differentially expressed. However, there were several GO categories that exceeded this 3.5 % threshold and are grouped according to their parent terms in Fig. 7. A more detailed flowchart of all GO terms can be found in Additional file 1: Figure S3. Of 16 genes annotated to the term “nuclear pore”, nine were differentially expressed, and all were downregulated. Of the 490 genes annotated to the term “structural constituent of ribosome”, 47 were differentially expressed, and of these 94 % were upregulated. All DE genes annotated as protease inhibitors were upregulated, including 8 of 19 genes encoding serine-type endopeptidase inhibitors, and 10 of 60 genes encoding peptidase inhibitor and regulator activity. Intracellular transport also appeared affected in the 764 samples, as 8 % annotated to non-membrane intracellular organelles were differentially expressed and most (82 %) were upregulated. All 10 DE genes encoding mitochondrial function were also upregulated. In addition, several genes (5 of 8) annotated with the biological process term “response to wounding” were upregulated. Taken together, these results suggested that protein synthesis was more active in the 764 seeds as compared to the nontransgenic controls. These results also suggest that aspects of intracellular transport and nuclear pore structures may be altered. The annotation of upregulated genes involved in wounding responses and peptidase inhibitors suggests that some aspects of a physical stress response may have been activated.

ST111 enriched GO terms were not as extensive as those found in the 764 line. However, it is of notable mention that 5 out of 29 genes (17.2 %) involving photosystem 1 were differentially expressed, all of them being downregulated. In addition, 4 out of 13 genes (31 %) were downregulated involving the photosystem 1 reaction center. Four out of 7 (43 %) detected phosphorylation genes were also differentially expressed and all were downregulated. In this case, it seems the ST111 line is exhibiting a reduction in metabolism and photosynthetic processes. The ST77 line being the most similar to wild type revealed no significant GO terms.

### Gene ontology results for 764 using AgriGO

Lists of all significantly differentially expressed genes as described above were exported to AgriGO for comparison with the *Glycine max* V2.1 GO background gene enrichment reference. Following analysis with AgriGO, the ST77 group again failed to show any highly significant GO term enrichment. ST111 samples show significant enrichment of photosynthesis and nucleic acid binding GO terms as reported by GOseq, while the 764 group did not. Likewise, the 764 group showed enriched terms indicating intracellular protein transport and translational terms, which were absent in the ST111 GO analysis. Overall, the results from GOseq and AgriGO were comparable with minor parent GO term variations. Complete AgriGO flow charts summarizing GO enrichment for the 764 line are shown in Additional file 1: Figure S3.

## Discussion

In this study, we addressed the possibility of detecting differentially expressed genes resulting from T-DNA insertion in three different transgenic soybean lines. Each line expressed and accumulated varying levels of recombinant protein targeted to seed tissue. Our experimental design allowed the testing of multiple factors that could potentially contribute to gene expression differences, including different recombinant proteins, progeny generation, and recombinant protein expression level. The inclusion of both edgeR and cufflinks allowed us to detect differentially expressed genes with high stringency while limiting false positives and characterize them using gene ontology enrichment analyses.

Contrary to our expectation that the transgenic line with the highest transgene or protein expression level would show the most drastic changes when compared to wild type, we found that the 764 line with moderate protein expression was the most different compared to wild type. Examination of transcript coverage across the T-DNA constructs shows an abrupt end in transcription before the end of the included terminator sequences, demonstrating tight transcription regulation and absence of non-terminated transcripts which have been reported from other transgenic soybean constructs utilizing the NOS terminator element [53]. In addition, line 764 lacks the tobacco etch virus enhancer element present in the other two lines, eliminating the possibility of downstream gene transcription effects [54]. This suggests that transcriptome alterations may not be fundamentally based on protein expression levels or insert complexity, but instead could be due to the attributes of the specific recombinant protein being expressed, mutations/disruptions from the insertion of T-DNA, or some combination of both. The hTG, mSEB, and hMBP-Sigma recombinant proteins all have very different physical characteristics, including size, charge, amino acid content and tertiary structure, therefore it is possible that accumulation of each recombinant protein could induce different response mechanisms within the seed. While 764 was not the highest expressing of the three transgenic lines, there may be characteristics of the mSEB protein that contributed to the observed transcriptome effects based on internal tolerance of the seed to this specific recombinant protein. Furthermore, while soybean has a relatively low mutation rate, mutations are commonly seen in plant tissue culture through transplantations and regeneration of tissues [55]. Alterations related to this are likely limited in their effects due to the generational distance of these lines from the initial transformation and the self-crossing nature of soybean limiting allelic variations. However, point mutations are still a possible occurrence that could potentially effect gene expression. Since this study focused on one specific mSEB event, it is unknown whether similar differential expression would be detected in other independent events transformed with the same 764 binary vector, or in 764 seed tissue derived from previous or subsequent generations. Indeed, gene expression responses to physical wounding from tissue culture procedures have been shown to carry over into the first generation of transformants [56], however the 764 line described here was harvested from fourth generation transgenic plants, limiting the potential for this kind of effect to contribute significantly to the extent of gene expression differences measured. Nonetheless, the possibility of random mutations occurring during propagation cannot be concluded to have no measureable effects.

In our datasets, we observed differential expression of helicase genes, suggesting the potential for DNA-level regulatory processes such as methylation, as well as down-regulation of genes for ribosomal subunits and translational processes in ST111 and 764. Furthermore, genes involved with transcriptional regulation and DNA/RNA binding are also differentially expressed consistent with potential gene silencing processes. Mapping the location of the transgene insert within the nuclear genome will reveal whether T-DNA integration has occurred in a transcriptionally active versus repressed region of the genome, and identify those genes (if any) that may have been disrupted as a result of the insertion, as past characterizations of T-DNA integrations in *Arabidopsis* demonstrated the capability of *Agrobacterium* to induce large deletions in genomic sequences [57, 58]. Information regarding neighboring genes in close proximity to the insert will allow exploration of methylation patterns, euchromatin and heterochromatin content of the integration site. The ST111 line will be of particular interest due to the relatively low expression and nearly absent transcript levels along the transgene open reading frame, suggesting the possibility of transcriptional level gene silencing which can occur in some events through methylation in the promoter region [59].

Post-translational regulation can also be a concern if it impacts recombinant protein turnover since decreased levels of accumulated protein could significantly impact downstream cost margins (e.g. of isolated therapeutics). The 764 line characterized in this study exhibited upregulation of serine-type endopeptidase inhibitors, which have been linked to delaying or reprogramming apoptotic processes [60–62]. Endopeptidase genes involving serine proteases in soybean seeds are typically upregulated as a response to tissue wounding or plant pathogen infections [62]. Serine proteases are also involved in proteolysis of the soybean β-conglycinin seed storage protein [63] in response to an increased demand for amino acids during translation [64]. The upregulation of genes involving translation and endopeptidase inhibitor activity in the 764 events suggests some induced response to programmed cell death (PCD) unrelated to a pathogenic infection. If recombinant protein accumulation activated endopeptidases as a result of PCD signals, then it is possible that the recombinant protein may become nicked, resulting in fragmented or degraded (e.g. undetectable) protein. We have previously noted endogenous nicking of recombinant mSEB protein in the 764 line [11], and other groups have also noted severe fragmentation of recombinant human growth hormone expressed in soy [65]. It should be noted that the seeds harvested and utilized in this study were fully matured and dried seeds in the R8 stage of maturation. Many genes expressed at this stage have been identified as proteases, ubiquitin and proteasome elements [66]. The products of these genes likely function in the elimination of proteins that are not necessary for seed germination processes. Likewise, mRNA transcripts relating to ribosomal machinery and transcription are upregulated in late seed development for immediate use during seed germination [67, 68]. It is possible that some of the differences in gene expression in line 764 are a result of delayed cessation of protein synthesis due to recombinant protein expression. If expression of the transgenes under control of the 7S or 11S promoters is extended, it may be possible to see the appearance of increased peptidase inhibitors as remnants of the cleanup phase following seed quiescence.

Gene ontology analysis allowed visualization of functional patterns of differentially expressed genes identified by both edgeR and cufflinks. No significant GO terms were identified from the list of ST77 differentially expressed genes, and only a few genes involved in photosynthesis and thylakoid functions were downregulated in ST111. However, the significant enrichment of GO terms related to translation and intercellular protein packaging and transport in 764 seeds shows a clear pattern in differentially expressed genes. Although the heterologous gene of interest is not present in the reference genome, the expression machinery utilized to synthesize and transport the seed-targeted protein is quantifiable, and is therefore detectable in our differential expression analyses as well as our GO enrichment analyses. Although it is unclear whether such genes are differentially expressed due to the expression of recombinant protein, but this may be one explanation for many of the GO terms observed involving intracellular transport and ribosomal constituents. It is also possible that small transcriptomic disruptions could have activated downstream cascades involved with gene regulation in a signaling type response to the initial disturbance via T-DNA insertion and position effects. Regardless, the upregulation of peptidase inhibitors is of potential concern if such a triggered response resulted from internal apoptotic signaling activated by the presence of high amounts of recombinant protein.

## Conclusions

The present study is a comparative analysis of differential gene expression in transgenic soybean seed tissue addressing multiple factors that could potentially induce changes in endogenous gene expression (e.g. transgene expression, protein accumulation, etc.). This study compared three separate transgenic lines expressing different recombinant proteins at varying levels, and found that all three lines exhibited differences in gene expression with one line (764) being substantially different. In this one line, nearly 10 % of the transcriptome was differentially expressed relative to wild type controls. Genes involving responses to wounding, translation, ribosomal constituents, endopeptidase inhibitors, cellular biosynthesis and gene expression were all upregulated while genes involving the nuclear envelope were downregulated. The results from this study suggest that the transcriptomic profiles of transgenic plants can be significantly different than those of wild type controls, and has provided a comprehensive first investigation into gene expression differences resulting from high levels of transgene expression and recombinant protein generation targeted to soybean seed tissues. It is not clear whether altered transcriptome profiles impact other variables traditionally characterized for the determination of substantial equivalence, though current literature suggests nutritional and metabolomic attributes remain comparable to non-transgenic plants. Based on the limited amount of differentially expressed genes shared between all three events, there doesn’t seem to be a consistent functional pattern induced based on transformation or recombinant protein expression, indicating each transformation event may respond differently to the inserted T-DNA or the resulting recombinant protein. As high throughput sequencing technologies advance and associated costs decrease, the selection of favorable transgenic lines based on transcriptome profiles could reveal valuable information beyond Mendelian breeding techniques and other methods currently used to characterize transgenic events. The approach proposed here can be utilized to investigate potential detrimental changes resulting from transgene integration and recombinant protein expression to maximize downstream recombinant protein yields in transgenic plants.

## Additional files

Additional file 1: Figure S3 AgriGO single enrichment analysis results for the 764 event. (**A**) Enriched biological process GO terms (**A**), cellular component GO terms (**B**), and molecular component GO terms (**C**) for 764. The gene list used as input was a merged list of all genes considered differentially expressed (DE) by edgeR and cufflinks with an FDR of 0.05. (TIFF 1136 kb) Additional file 2: Table S1 Samples ligated to Illumina TruSeq adapters and their respective sequences. The specific lanes in which samples were loaded on the Illumina flow cell are indicated, as well as total reads, mapped reads, single and multi-mapping reads, percent mapping reads, and percent multimapping reads per sample. (TIFF 456 kb) Additional file 3: Figure S1 Multi-dimensional scaling plots of variance between samples from edgeR. Sample variance between ST77 (**A**), ST111 (**B**), and 764 (**C**) versus wild type are plotted based on differentially expressed gene number and fold change. The cluster dendrograms include all expressed genes for ST77 (**D**), ST111 (**E**) and 764 (**F**), showing the Euclidean distance between each sample based on overall gene expression. (TIFF 789 kb) Additional file 4: Figure S2 Normalization curves of gene density from cummeRbund of each transgenic event versus wild type (**A**, **C**, **E**) and each sample (**B**, **D**, **F**). (TIFF 2282 kb)

## Abbreviations

764: Transgenic soybean line expressing nontoxic *Staphylococcus* enterotoxin B
DE: Differentially expressed
GO: Gene ontology
hMBP: Human myelin basic protein
hTG: Human thyroglobulin
mSEB: Non-toxic mutant *Staphylococcus* enterotoxin B
PCD: Programmed cell death
ST77: Transgenic soybean line expressing human thyroglobulin
ST111: Transgenic soybean line expressing human myelin basic protein
T-DNA: Transfer DNA
TSP: Total soluble protein
VSP: Vegetative storage protein
