# Supplementary figures and images for: A Comparison of transgenic and wild type soybean seeds: analysis of transcriptome profiles using RNA-Seq
Source: BMC Biotechnol. 2015 Oct 1;15:89. doi: 10.1186/s12896-015-0207-z (PMC4591623; doi:10.1186/s12896-015-0207-z)

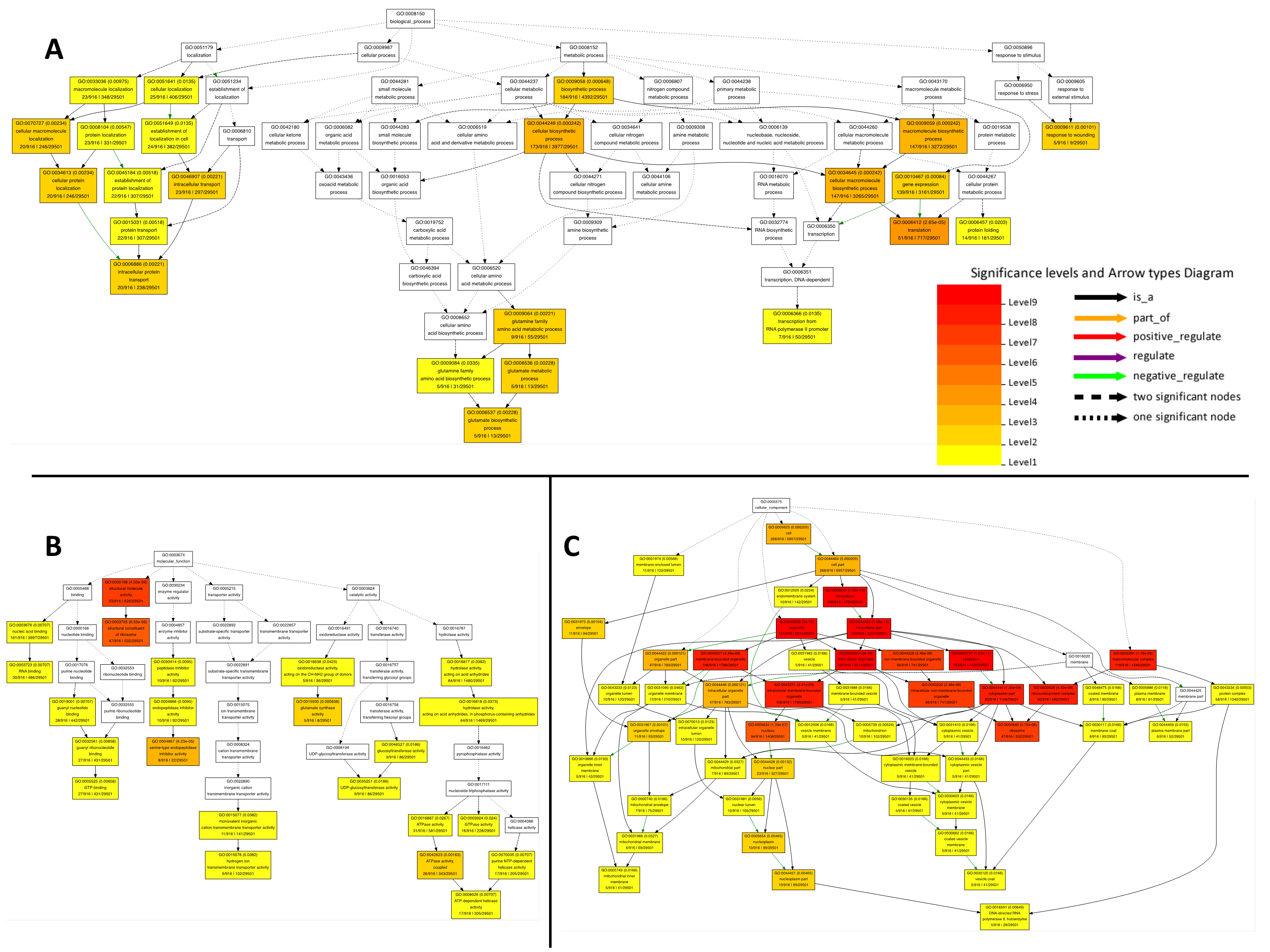

Supplement: Additional file 1: Figure S3 — AgriGO single enrichment analysis results for the 764 event. (A) Enriched biological process GO terms (A), cellular component GO terms (B), and molecular component GO terms (C) for 764. The gene list used as input was a merged list of all genes considered differentially expressed (DE) by edgeR and cufflinks with an FDR of 0.05. (TIFF 1136 kb) [file 12896_2015_207_MOESM1_ESM.tif]

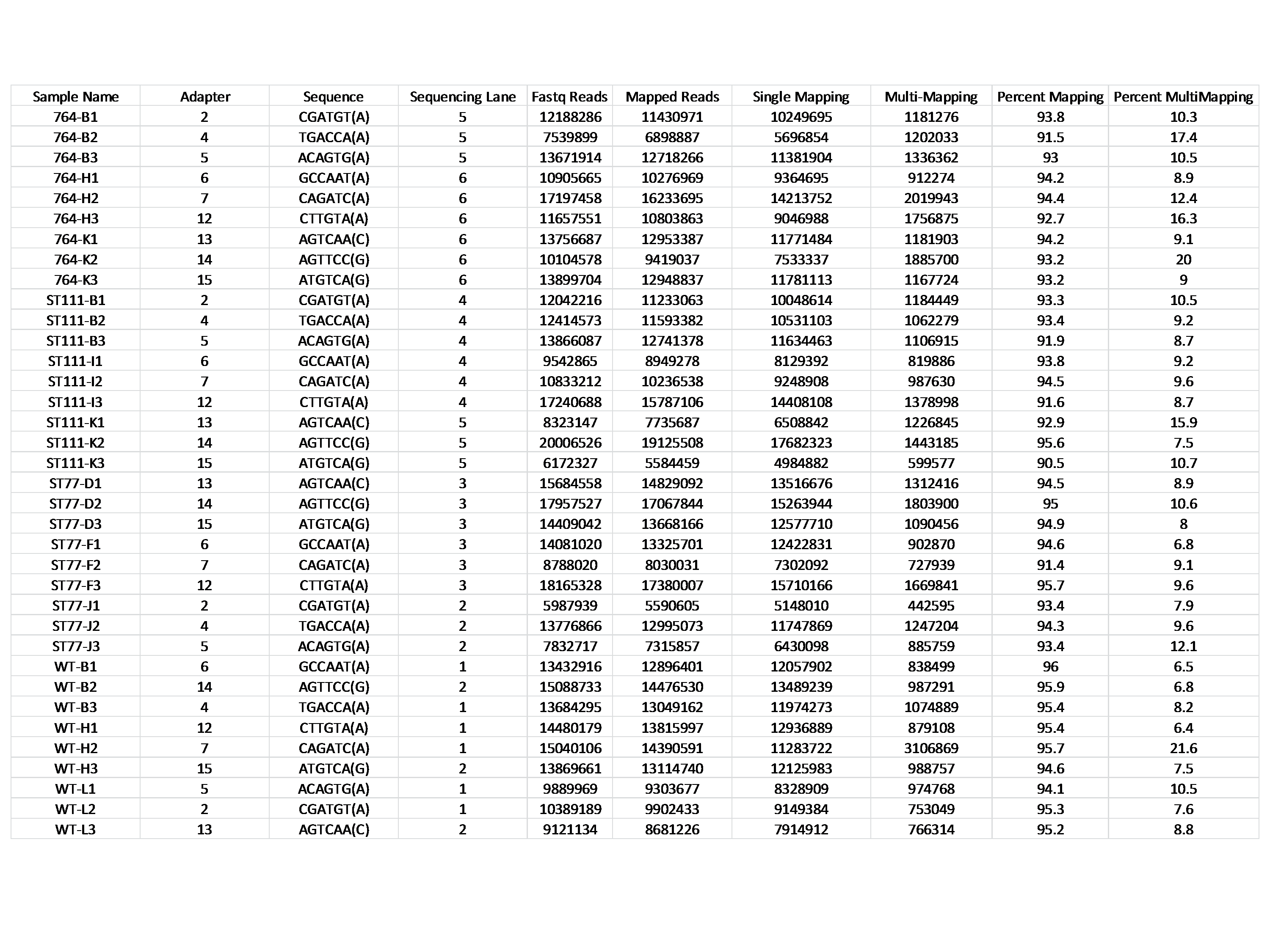

Supplement: Additional file 2: Table S1 — Samples ligated to Illumina TruSeq adapters and their respective sequences. The specific lanes in which samples were loaded on the Illumina flow cell are indicated, as well as total reads, mapped reads, single and multi-mapping reads, percent mapping reads, and percent multimapping reads per sample. (TIFF 456 kb) [file 12896_2015_207_MOESM2_ESM.tif]

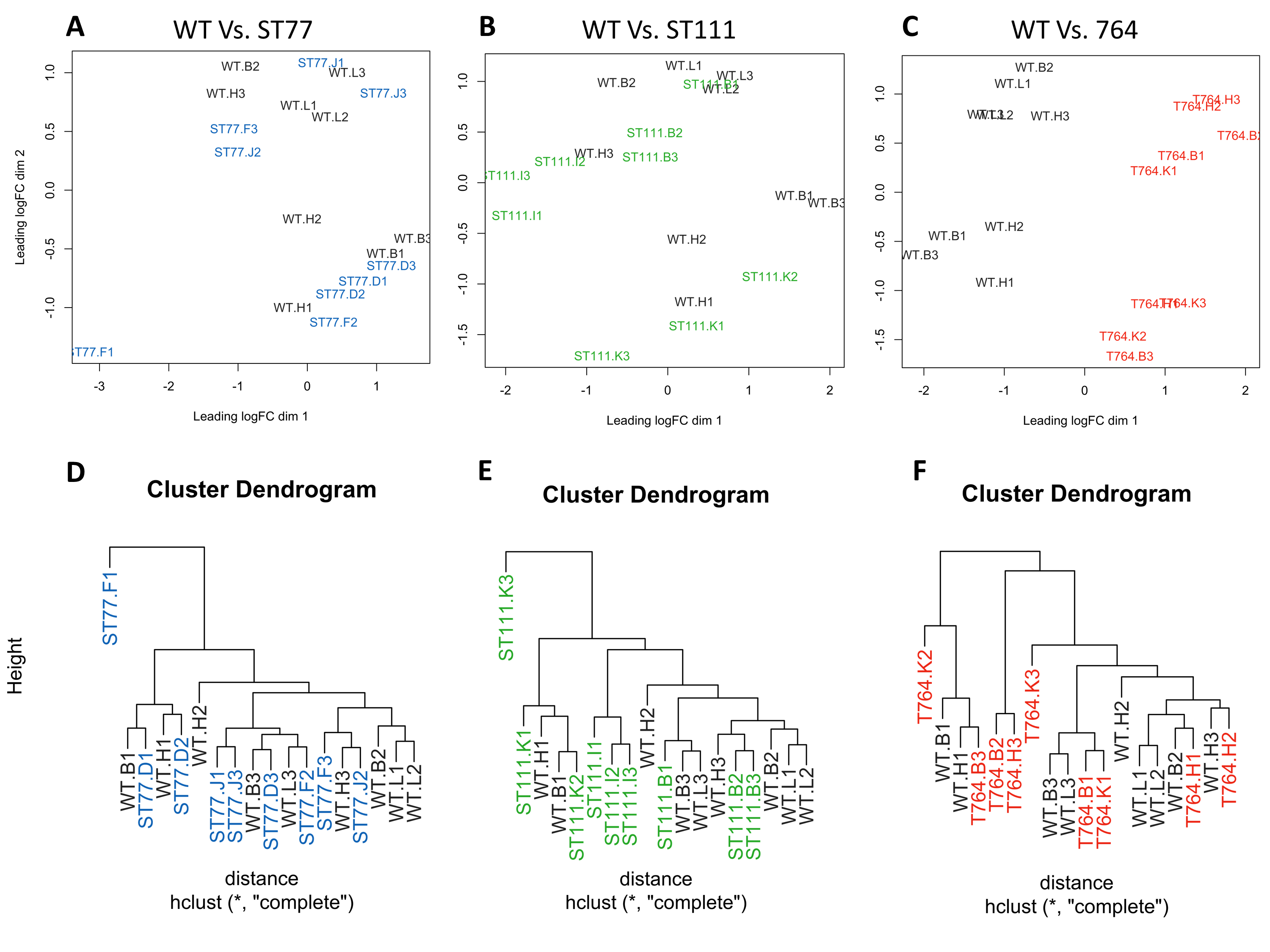

Supplement: Additional file 3: Figure S1 — Multi-dimensional scaling plots of variance between samples from edgeR. Sample variance between ST77 (A), ST111 (B), and 764 (C) versus wild type are plotted based on differentially expressed gene number and fold change. The cluster dendrograms include all expressed genes for ST77 (D), ST111 (E) and 764 (F), showing the Euclidean distance between each sample based on overall gene expression. (TIFF 789 kb) [file 12896_2015_207_MOESM3_ESM.tif]

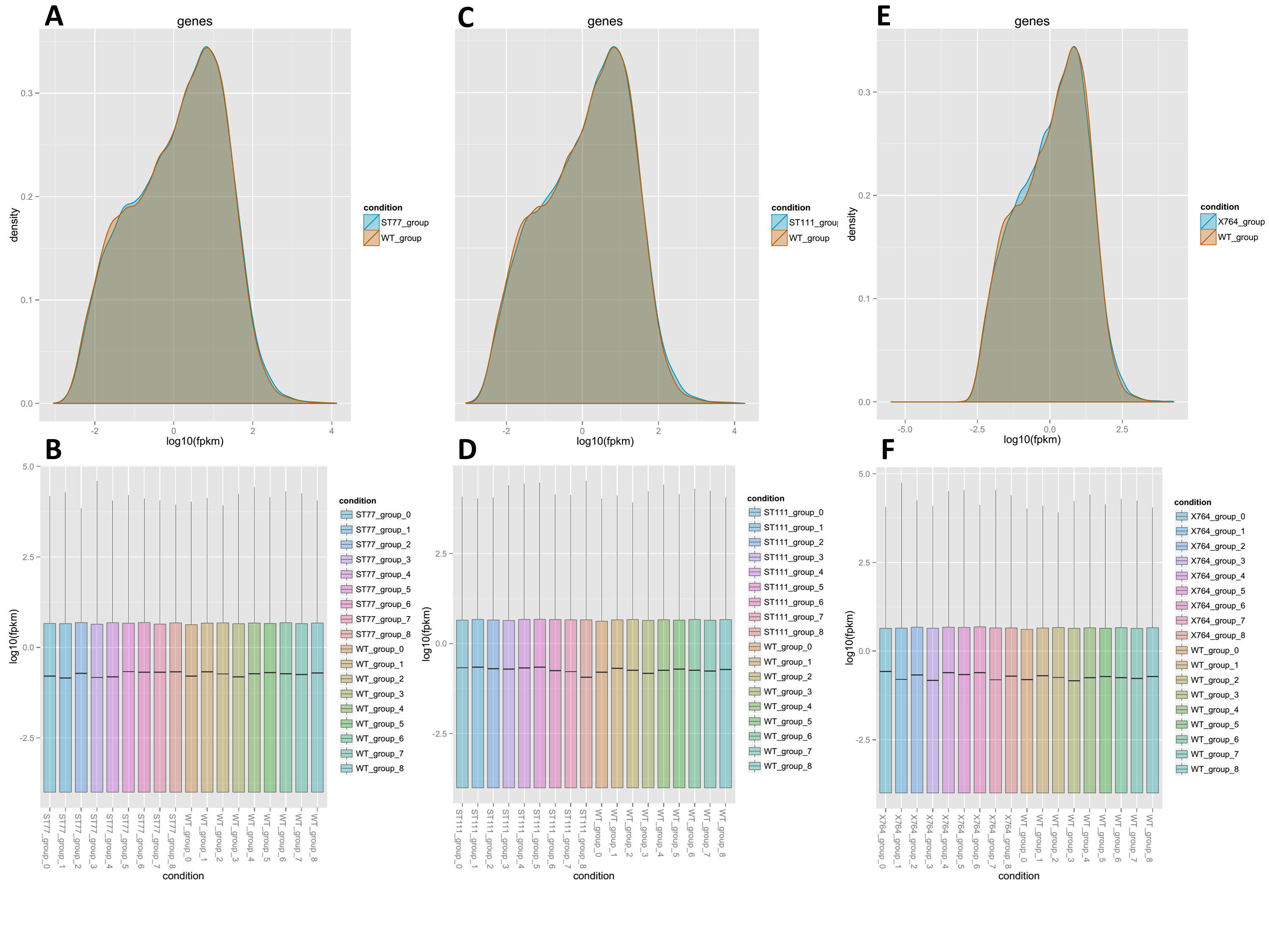

Supplement: Additional file 4: Figure S2 — Normalization curves of gene density from cummeRbund of each transgenic event versus wild type (A, C, E) and each sample (B, D, F). (TIFF 2282 kb) [file 12896_2015_207_MOESM4_ESM.tif]
